# Ethanol Ablation of a Thyroglossal Duct Cyst and Review of the Literature

**DOI:** 10.1210/jcemcr/luad070

**Published:** 2023-06-28

**Authors:** Mason D Stillman, Nicholas A Waring, Jennifer H Kuo

**Affiliations:** Section of Endocrine Surgery, Columbia University, New York, NY 10032, USA; Section of Endocrine Surgery, Columbia University, New York, NY 10032, USA; Section of Endocrine Surgery, Columbia University, New York, NY 10032, USA

**Keywords:** ethanol ablation, thyroglossal duct cyst

## Abstract

Ultrasound-guided ethanol ablation (EA) is a less invasive alternative to surgical resection for the management of thyroglossal duct cysts (TGDCs). However, to date, EA is rarely used in the United States to treat TGDCs. We present a case of TGDC successfully treated with EA in the United States. A 66-year-old man presented with a mobile anterior neck mass. Neck ultrasonography revealed a complex cystic mass in the midline directly anterior to the trachea, measuring 52 × 41 × 50 mm. Fine needle aspiration revealed no malignant cells, and pathology was consistent with TGDC. The patient had no contraindications to surgical resection. The patient's pretreatment symptom score was 7 and cosmetic score was 3. One month after EA, volume reduction ratio was 40%, symptom score was 1, and cosmetic score was 3. Four months after EA, the TGDC was resolved without need for an additional procedure. The volume reduction ratio was 96.8%, and symptom score and cosmetic score were both 1. In summary, EA is a viable alternative to surgical resection, even in patients who are surgical candidates. EA is attractive due to its simplicity, cost effectiveness, and tolerable side effect profile. Further studies are needed to evaluate long-term safety and efficacy, particularly in United States patients.

## Introduction

Thyroglossal duct cysts (TGDCs) are the most common congenital cyst of the neck, consisting of epithelial remnants of the thyroglossal duct. TGDCs are benign and present clinically as a mobile midline anterior neck mass. Treatment is advised to exclude malignancy and prevent recurrent infection. Standard of care for management of TGDCs is the Sistrunk procedure, which entails surgical resection of the cyst along with the midportion of the hyoid bone and a core of tissue from the hyoid bone to the foramen cecum.

Another, less invasive treatment option for TGDCs is ultrasound-guided ethanol ablation (EA). EA is currently rarely used for TGDC in the United States; however, it has been used successfully in Europe and Asia for the last decade, with increasing frequency [1-7, 10]. In this case report we describe the successful treatment of a TGDC with EA at our institution in the United States.

## Case Presentation

A 66-year-old man with a history of hypertension, hyperlipidemia, and psoriatic arthritis presented for evaluation with an enlarging neck mass following an upper respiratory infection. He denied any dysphagia, dyspnea, dysphonia, or any history of radiation. However, due to the large size of the mass, he noted increased neck pressure, and was significantly bothered by the cosmesis of the mass. He had no past thyroid history but a positive family history of Hashimoto thyroiditis in his son (33 years old). On examination (conducted via video visit) the patient had a visible midline mass inferior to the mandible that was mobile with deglutition. His thyroid stimulating hormone level was 2.68 µIU/mL (mIU/L) and serum calcium was 9.6 mg/dL (2.4 mmol/L).

**Figure 1.**
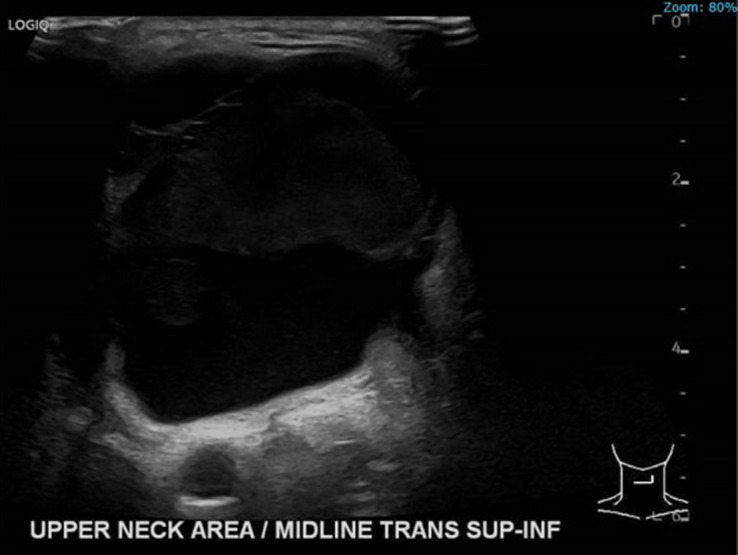
Neck ultrasonography showing thyroglossal duct cyst prior to ethanol ablation.

Neck ultrasonography performed in clinic by the patient's endocrinologist revealed a complex cystic mass in the midline directly anterior to the trachea and above the thyroid cartilage, measuring 52 × 41 × 50 mm (Fig. 1). The patient was then referred to Dr. Kuo for surgical evaluation. Subsequent ultrasonography showed a large cystic and septated TGDC, measuring 46 × 48 × 32 mm. Fine needle aspiration revealed no malignant cells, a few groups of benign columnar cells, histiocyte-like cells, cholesterol crystals, blood, and inflammatory cells, consistent with TGDC.

## Diagnostic Assessment

The patient had no contraindications to surgical resection but had strong reservations about the Sistrunk procedure, which typically removes a piece of the hyoid bone, and researched nonsurgical interventions. Given the cystic nature of the TGDC, he wanted to pursue EA first and would be amenable to surgical resection if EA failed. He was given the option to undergo Sistrunk procedure vs ultrasound-guided EA, and he elected to undergo EA. His pretreatment cosmetic score was 3 (grade 1, no palpable mass; grade 2, invisible but palpable mass; grade 3, mass visible only to an experienced clinician; grade 4, easily visible mass) His pretreatment symptom score was 7 (a visual analog scale rating pressure symptoms from 1 to 10).

## Treatment

A 16-gauge needle was inserted into the cyst, and approximately 36 mL of dark, hemorrhagic fluid was aspirated. The cyst was thoroughly irrigated with cold sterile saline repeatedly until the fluid cleared and then irrigated continuously with 100% ethanol until the fluid became cloudy. Finally, 7 mL of 100% ethanol were infused. The patient tolerated the procedure without any voice change and with minimal pain.

## Outcome and Follow-up

**Figure 2.**
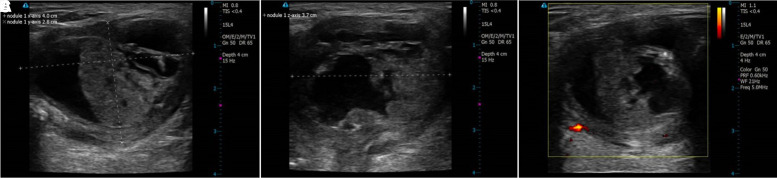
Neck ultrasonography 1 month after ethanol ablation. A and B, transverse and longitudinal views. C, Doppler ultrasound.

**Figure 3.**
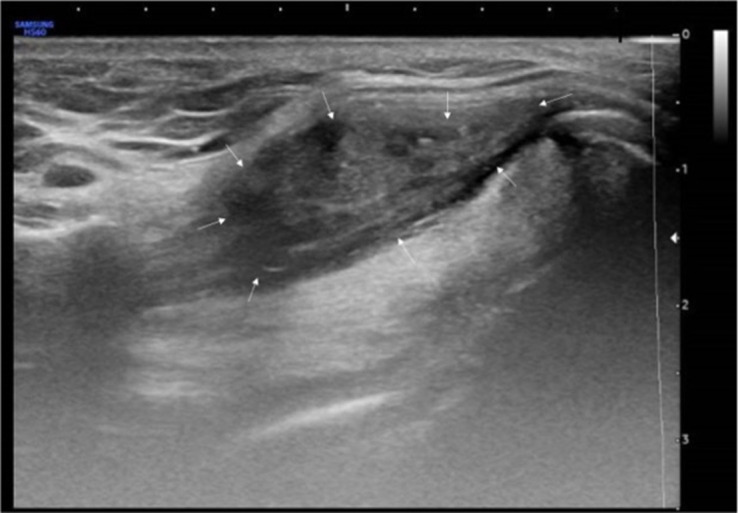
Neck ultrasonography 4 months after ethanol ablation; cystic component almost completely resolved.

**Figure 4.**
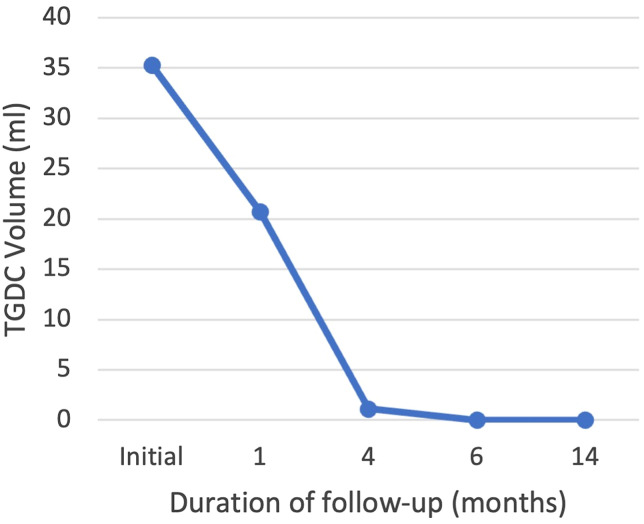
Thyroglossal duct cyst volume over 14 months of follow-up.

One month after undergoing EA, follow-up ultrasonography showed a mixed composition thyroglossal duct cyst measuring 40 × 28 × 37 mm with an anechoic lateral-superior aspect and isoechoic solid portion more medially (Fig. 2). Volume ratio reduction (VRR) was 41.3%, symptom score was 1, and cosmetic score was 3. At 4 months post procedure, ultrasonography showed a residual TGDC measuring 16 × 14 × 10 mm with the cystic component almost completely resolved leaving a hypoechoic solid component (Fig. 3). VRR was 96.9%, and symptom score and cosmetic score were both 1. The patient felt the cyst was noticeably smaller and reported resolution of his symptoms. One-year follow-up ultrasonography revealed complete resolution of the TGDC. TGDC volume over the duration of follow-up is depicted in Fig. 4.

## Discussion

Percutaneous ethanol instillation is a form of chemical ablation that has been well studied in the treatment of hepatocellular carcinoma for patients with cirrhosis, as well as more recently in the treatment of benign thyroid cysts. Diffusion of ethanol into cystic epithelial cells dehydrates the cytoplasm and denatures proteins, resulting in coagulative necrosis of the cyst wall. Subsequent diffusion of ethanol into local circulation causes vascular endothelial necrosis, platelet aggregation and thrombosis, and ischemic necrosis of surrounding tissues. Chemical ablation is an attractive option due to its simplicity, cost effectiveness, and tolerable side effect profile, which includes transient fever, pain, swelling, hoarseness, and dysphagia.

Recent studies comprising fewer than 200 patients in Korea, Hong Kong, and Turkey have demonstrated mean VRRs ranging from 73% to 96.2% at final follow-up examination [1-7, 10]. The largest among them was performed by Park et al in Korea, with 77 patients undergoing EA for TGDC. Mean index cyst volume was 7.3 mL, and mean VRR at 3 months of follow-up was 73% ± 31%. Long-term success was achieved in 35 of 42 patients with follow-up data longer than 2 years. At a median follow-up period of 69 months, mean VRR was 81% ± 35% [2]. Case series of patients with TGDC treated with EA are summarized in Table 1.

**Table 1.** Studies of ethanol ablation for thyroglossal duct cyst

Study	Country	Number of patients	Index cyst volume (mL)	VRR (mean)	Follow-up period
**Baskin (2006)** [8]	US	3	N/R	N/R*^a^*	3-5 months
**Kim (2011) [**5]	Korea	11	6.0 (mean)	81.3%	3-29 months
**Chow (2012)** [4]	Korea	6	3.5 (median)	N/R*^b^*	13-72 months
**Lee (2015)** [3]	Hong Kong	9	8.9 (mean)	76.6%	12-15 months
**Chung (2017)** [6]	Korea	56	3.9 (median)	82.3%	3-12 months
**Karatay (2021)** [1]	Turkey	28	4.1 (median)	95.1%	12 months
**Lee (2021)** [9]	US	8	10.6 *^c^* (mean)	74.4%*^c^*	3 months
**Park (2021)** [7]	Korea	82	N/R	84%*^d^*	10-34.5 months
**Park (2022)** [2]	Korea	77	7.3 (mean)	73%	3 months
**Ahn (2023)** [10]	Korea	28	6.7 (median)	96.2% (median)	12 months

a 1/3 patients experienced treatment success, VRR not reported.

b 5/6 patients experienced treatment success, VRR not reported.

c Figure reflects all cystic lesions treated (n = 25), not limited to TGDC.

d % Patients who experienced >50% volume reduction, VRR not reported.

A recent meta-analysis compared the efficacy of EA to OK-432 sclerotherapy in 129 patients with TGDC. The success rate, defined as VRR >50%, was found to be superior in the EA group compared to that of OK-432, although with equivocal statistical significance (84% vs 51%, *P* = .055). No major complications occurred in the ethanol group (n = 82), and only one major complication of laryngeal edema occurred in the OK-432 group (n = 47) 2 days after the procedure and was successfully treated with steroids [7].

In comparison to surgery, EA has better short-term morbidity but higher likelihood of treatment failure. A recent study from Korea including 345 patients (289 surgery, 56 EA) found that EA demonstrated fewer complications compared to Sistrunk operation (1.8% vs 10.0%, *P* = .04) but higher rates of treatment failure (19.6% vs 2.4%, *P* < .001). In addition, EA was 3.4 times less expensive compared to surgery [6].

It should be noted that the vast majority of published cases of TGDC treated with EA occurred outside the United States. One report exists of 3 patients with TGDC treated with EA in Florida in 2006. In that report, 2 of the 3 patients experienced cyst recurrence [8]. One other series of 8 patients was published in 2021. Mean index cyst volume was 10.6 mL, and mean VRR at 3 months follow-up was 74.4%, although these figures include other benign head and neck cystic lesions besides TGDC (n = 25) [9]. To our knowledge, no other reports exist of EA for TGDC exist on patients in the United States. Accordingly, our case represents a valuable addition to the literature.

Our patient tolerated the procedure well with minimal pain or discomfort. At his 4-month follow-up evaluation, his VRR was 96.8%, with symptom and cosmetic scores of 1. Ultrasonography demonstrated almost complete resolution of the cyst, with only a small, solid, hypoechoic portion remaining. A VRR of 96.8% represents an excellent response to therapy, greater than the mean of all studies in Table 1 [1-10].

## Learning Points

- EA may be a safe and viable alternative to surgery for the treatment of TGDC and may be considered as a first-line therapy in patients wishing to avoid surgery.
- EA has a greater likelihood of recurrence compared with surgery but has fewer complications.
- EA is simple, cost-effective, and has a tolerable side effect profile.

## Contributors

M.D.S. and N.A.W. drafted the manuscript. J.H.K. performed diagnostic and surgical procedures, patient counseling, and editing of the manuscript. The authors read and approved the final manuscript.

## Data Availability

Data sharing is not applicable to this article as no datasets were generated or analyzed during the current study.

## Abbreviations

EA: ethanol ablation
TGDC: thyroglossal duct cyst
VRR: volume ratio reduction

## References

[1] Karatay E, Javadov M. The effectiveness of ethanol ablation in the treatment of thyroglossal duct cysts in adult cases and evaluation with cosmetic scoring. Jpn J Radiol. 2021;39(10):994‐999. 33993431 10.1007/s11604-021-01135-3

[2] Park SI, Baek JH, Chung SR, et al Ethanol ablation for the treatment of thyroglossal duct cysts: follow-up results for longer than 2 years. Eur Radiol. 2022;32(5):3525‐3531. 34993573 10.1007/s00330-021-08402-x

[3] Lee DK, Seo JW, Park HS, et al Efficacy of ethanol ablation for thyroglossal duct cyst. Ann Otol Rhinol Laryngol. 2015;124(1):62‐67. 25048959 10.1177/0003489414542845

[4] Chow TL, Choi CY, Hui JYH. Thyroglossal duct cysts in adults treated by ethanol sclerotherapy: a pilot study of a nonsurgical technique. Laryngoscope. 2012;122(6):1262‐1264. 22461135 10.1002/lary.23254

[5] Kim SM, Baek JH, Kim YS, et al Efficacy and safety of ethanol ablation for thyroglossal duct cysts. AJNR Am J Neuroradiol. 2011;32(2):306‐309. 21087937 10.3174/ajnr.A2296 PMC7965726

[6] Chung MS, Baek JH, Lee JH, et al Treatment efficacy and safety of ethanol ablation for thyroglossal duct cysts: a comparison with surgery. Eur Radiol. 2017;27(7):2708‐2716. 27957639 10.1007/s00330-016-4659-x

[7] Park SI, Baek JH, Suh CH, et al Chemical ablation using ethanol or OK-432 for the treatment of thyroglossal duct cysts: a systematic review and meta-analysis. Eur Radiol. 2021;31(12):9048‐9056. 34003346 10.1007/s00330-021-08033-2

[8] Baskin HJ. Percutaneous ethanol injection of thyroglossal duct cysts. Endocr Pract. 2006;12(4):355‐357. 16939947 10.4158/EP.12.4.355

[9] Lee E, Park I, Elzomor A, et al Efficacy of ethanol ablation as a treatment of benign head and neck cystic lesions. Am J Otolaryngol. 2021;42(6):103082. 10.1016/j.amjoto.2021.103082 34029918

[10] Ahn D, Kwak JH, Lee GJ, Sohn JH. Ultrasound-guided ethanol ablation as a primary treatment for thyroglossal duct cyst: feasibility, characteristics, and outcomes. Otolaryngol Neck Surg. 2023;168(6):1381‐1388. 10.1002/ohn.231 36939631

